# Supplementary material for: MetaRibo-Seq measures translation in microbiomes
Source: Nat Commun. 2020 Jun 29;11:3268. doi: 10.1038/s41467-020-17081-z (PMC7324362; doi:10.1038/s41467-020-17081-z)
Supplement: Supplementary file 10 — Supplementary Data 7 [file 41467_2020_17081_MOESM10_ESM.zip › File2/Confidence_High_Taxonomy/135046_out.krona.html]

Javascript must be enabled to view this page.

members
magnitude
magnitudeUnassigned
count
unassigned
taxon
rank

135046\_out

20

2
20
superkingdom

phylum
976
20

class
20
200643

171549
20
order

family
815
20

genus
816
20


SRS017622\_contig\_number\_35
species
1
2292956

species

SRS014613\_contig\_number\_884SRS015264\_contig\_number\_9264SRS016132\_contig\_number\_5515SRS016267\_contig\_number\_11558SRS016495\_contig\_number\_274SRS018313\_contig\_number\_10196SRS019267\_contig\_number\_6630SRS019787\_contig\_number\_12078SRS024132\_contig\_number\_43092SRS043701\_contig\_number\_14655SRS050299\_contig\_number\_10400SRS053573\_contig\_number\_2022SRS064276\_contig\_number\_34607SRS078419\_contig\_number\_29860SRS1041145\_contig\_number\_33243SRS143342\_contig\_number\_19729SRS144135\_contig\_number\_contig-100\_1389.172442SRS146812\_contig\_number\_59327SRS149075\_contig\_number\_1119
19
817
